# Azithromycin (Zithromax^®^)

**DOI:** 10.1155/S1064744996000415

**Published:** 1996

**Authors:** James M. McCarty

**Affiliations:** Hilltop Research Centers 6079 N. Fresno #101 Fresno CA 93710 USA

## Abstract

Azithromycin (Zithromax^®^, Pfizer, Inc., New York, NY) is a 15-membered-ring macrolide and the first azalide antibiotic. It is distinguished from other macrolides by its rapid and extensive penetration into intracellular and interstitial tissue compartments, accompanied by prolonged tissue and serum half-lives. Azithromycin shares the gram-positive activity of erythromycin but is more potent against gram-negative organisms. For urethritis and cervicitis caused by *Chlamydia trachomatis*, azithromycin is effective and well tolerated in a single dose of 1 g, a regimen recommended by the CDC. A 5-day dosage regimen is available for the treatment of community-acquired respiratory-tract and skin and skin-structure infections caused by susceptible organisms. Azithromycin provides short-duration, high-compliance, cost-effective regimens that should improve outcomes.


Infectious Diseases in Obstetrics and Gynecology 4:215–220 (1996)
© 1997 Wiley-Liss, Inc.

### Azithromycin (Zithromax®)

James M. McCarty
*Hilltop Research Centers, Fresno, CA*

ABSTRACT

**Azithromycin (Zithromax®, Pfizer, Inc., New York, NY) is a 15-membered-ring macrolide and the first azalide antibiotic. It is distinguished from other macrolides by its rapid and extensive penetration into intracellular and interstitial tissue compartments, accompanied by prolonged tissue and serum half-lives. Azithromycin shares the gram-positive activity of erythromycin but is more potent against gram-negative organisms. For urethritis and cervicitis caused by *Chlamydia trachomatis,* azithromycin is effective and well tolerated in a single dose of 1 g, a regimen recommended by the CDC. A 5-day dosage regimen is available for the treatment of community-acquired respiratory-tract and skin and skin-structure infections caused by susceptible organisms. Azithromycin provides short-duration, high-compliance, cost-effective regimens that should improve outcomes.** © 1997 Wiley-Liss, Inc.

KEY WORDS
Azalide, urethritis, cervicitis, *Chlamydia trachomatis*

Azithromycin (Zithromax®, Pfizer, Inc., New York, NY), the first azalide antibiotic, has been approved for the single-dose treatment of nongonococcal urethritis and cervicitis due to *Chlamydia trachomatis*. In addition, azithromycin is useful in a 5-day regimen for the treatment of indicated respiratory-tract and skin and skin-structure infections (Table 1) (unpublished data, Pfizer, Inc., 1996).

*C. trachomatis* is the most common sexually transmitted pathogen in the United States.[1] An obligate intracellular parasite,[2] *C. trachomatis* is the leading cause of nongonococcal urethritis and epididymitis in men under 35 years of age. In women, it causes mucopurulent cervicitis, urethral syndrome, and pelvic inflammatory disease (PID).[1]

The health-care costs of *C. trachomatis* infections are substantial.[3] In 1990, the direct and indirect costs incurred with PID and PID-associated ectopic pregnancy and infertility—medical problems frequently arising from untreated, uncomplicated chlamydial infections—were estimated to be $4.2 billion.[4]

The treatment of genital chlamydial infections has been difficult. Tetracycline or doxycycline administered orally for 7 days has been widely prescribed as a first-line agent for chlamydial urethritis and cervicitis,[2] although the treatment is limited by mild, bothersome side effects and frequent dosing that can result in poor compliance.[5] In addition, both tetracycline and doxycycline are contraindicated in pregnant patients.[6] Among the macrolides, erythromycin has been used in pregnant women and in patients intolerant of tetracyclines, although it has been poorly tolerated in dosages shown to be clinically effective against *C. trachomatis* infection.[1,3]

### CHEMISTRY

Azithromycin is a 15-membered-ring macrolide[7] that differs from erythromycin by the presence of a methyl-substituted nitrogen in the macrolide ring.

**Address correspondence/reprint requests to Dr. James M. McCarty, Hilltop Research Centers, 6079 N. Fresno #101, Fresno CA 93710.**

Received July 2, 1996
Accepted July 16, 1996

TABLE 1. Approved indications for azithromycin

| Infection | Pathogens | Dosage |
|---|---|---|
| Nongonococcal urethritis and cervicitis | *Chlamydia trachomatis* | 1 g × 1 |
| Acute bacterial exacerbation of chronic obstructive pulmonary disease | *Streptococcus pneumoniae*<br>*Haemophilus influenzae*<br>*Moraxella catarrhalis* | 500 mg × 1 day, 250 mg × 4 days |
| Community-acquired pneumonia[a] | *S. pneumoniae*<br>*H. influenzae* | 500 mg × 1 day, 250 mg × 4 days |
| Pharyngitis and tonsillitis[b] | *S. pyogenes* | 500 mg × 1 day, 250 mg × 4 days (adults)<br>12 mg/kg × 5 days (children) |
| Acute otitis media | *S. pneumoniae*<br>*H. influenzae*<br>*M. catarrhalis* | 10 mg/kg × 1 day, 5 mg/kg × 4 days (children) |
| Uncomplicated skin and skin-structure infections | *Staphylococcus aureus*<br>*S. pyogenes*<br>*S. agalactiae* | 500 mg × 1 day, 250 mg × 4 days |

[a]Outpatient pneumonia of mild severity.
[b]Penicillin is first-line therapy.

In contrast, clarithromycin and dirithromycin are 14-membered ring macrolides that are analogs of erythromycin.[7,8]

### MECHANISM OF ACTION

Azithromycin, like erythromycin, produces its antibacterial effects by inhibiting bacterial protein synthesis through binding to the 50S ribosomal subunit of susceptible organisms.[9]

### PHARMACOKINETICS

Azithromycin is distinguished from erythromycin by its unique cellular kinetics, which include rapid and extensive penetration into intracellular and interstitial tissue compartments and high and sustained tissue levels accompanied by relatively low serum levels. The absorption of azithromycin is generally more predictable than that of erythromycin, producing higher tissue and intracellular concentrations.[8,10,11] After absorption, azithromycin is distributed rapidly from serum.[7]

The bioavailability of azithromycin is 37%.[12] The elimination half-life [36–40 h, as determined 24–72 h after a 500-mg intravenous (IV) dose] is more than 10-fold that of other available macrolides.[7,12]

The concentration of azithromycin in most tissues exceeds that in serum by 10- to 100-fold.[12] The projected tissue levels in the prostate or uterus following a single 1-g dose remain well above the minimum inhibitory concentration for 90% ($MIC_{90}$) of *C. trachomatis* isolates for as long as 10 days (Fig. 1).[12,13] In women who received a single 500-mg oral dose of azithromycin 24–96 h before surgery, high drug concentrations were found in gynecologic tissue up to 96 h after administration.[14] The mean maximum concentration 24 h after dosing was 1.44 μg/g, and the estimated tissue half-life was 67 h. The high, sustained concentrations of azithromycin in gynecologic tissue result in the presence of drug above the $MIC_{90}$ throughout the long life cycle of *C. trachomatis.*

Delivery by phagocytes enhances the concentrations of azithromycin at sites of infection. The uptake by polymorphonuclear leukocytes (PMLs) and alveolar or peritoneal macrophages is rapid.[15] After 2 h of incubation, the intracellular or extracellular concentration ratio was 79 in human PMLs and 62 in murine peritoneal macrophages, compared with erythromycin ratios of 16 and 4, respectively. After 24 h, azithromycin was concentrated 10-fold higher than erythromycin in human PMLs and 26-fold higher in murine peritoneal macrophages. One hour after the removal of extracellular erythromycin, 85% of the accumulated stores were released. In contrast, the removal of extracellular azithromycin resulted in a slow release from intracellular stores (only 19% after 1 h). The exposure of the cells to bacteria enhanced the release of azithromycin from phagocytes.

The significance of the sustained tissue and intracellular concentrations of azithromycin is reflected in its dosage schedule. Azithromycin is administered in a single 1-g dose to patients with uncomplicated urethritis or cervicitis due to *C. tra-*

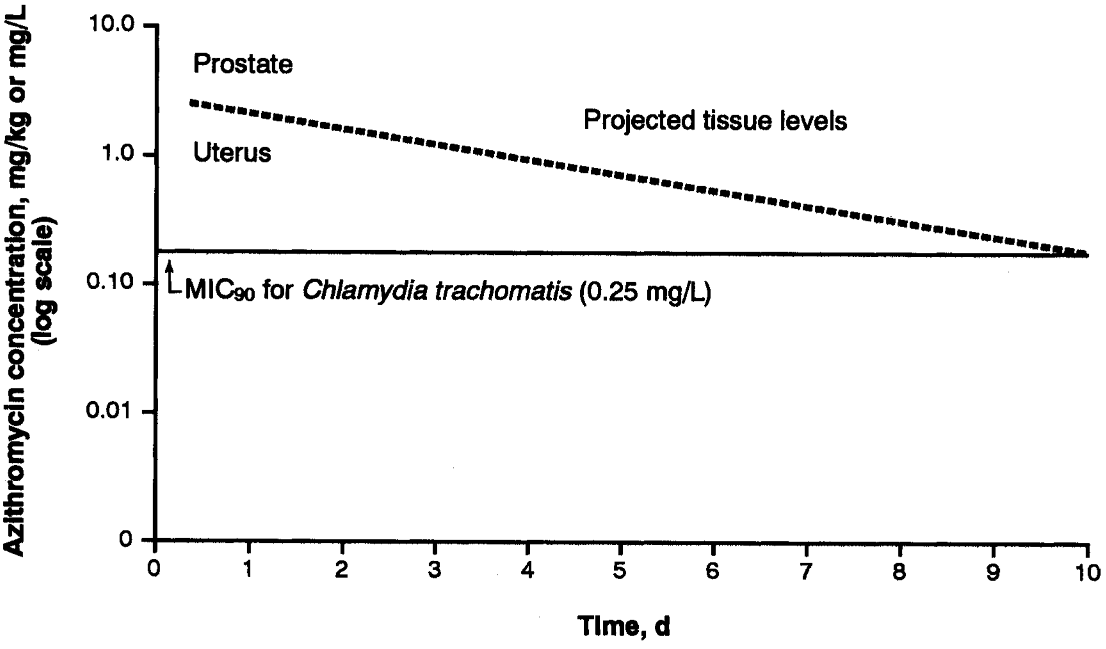

Fig. 1. Projected tissue levels of a single 1-g dose of azithromycin over 10 days (adapted from Foulds et al.[12] and Scieux et al.[13]).

*chomatis*. For adults with indicated respiratory-tract or skin and skin-structure infections (see below), azithromycin is administered on a 5-day dosage schedule—500 mg on day 1, followed by 250 mg once daily on days 2–5 (unpublished data, Pfizer, Inc., 1996).

Azithromycin is also supplied as a powder for pediatric oral suspension (unpublished data, Pfizer, Inc., 1996). When administered at 10 mg/kg on day 1 followed by 5 mg/kg on days 2–5 in children 1–15 years old, the mean pharmacokinetic parameters at day 5 were comparable to those seen in adults. Food increases the rate of absorption of azithromycin given as the pediatric oral suspension, whereas the extent of absorption is unchanged. Food decreases the absorption of the capsule formulation of azithromycin. Both capsules and the oral suspension should be given at least 1 h before or 2 h after a meal.

### SIDE EFFECTS

Azithromycin shares the favorable overall safety profile of erythromycin[8] but demonstrates better gastrointestinal tolerability. Among 2,922 adults scheduled to receive multiple-dose azithromycin in clinical trials, the treatment was discontinued because of side effects—chiefly gastrointestinal symptoms—in 22 patients (0.8%), significantly fewer than in erythromcyin recipients (24/450, 5.3%) or in those receiving any comparative agent (72/2,835, 2.5%).[16] The incidence of side effects in children was comparable to that in adults.

The most common side effects in adults receiving a single 1-g dose of azithromycin involved the gastrointestinal system. The side effects that occurred with a frequency of ≥1% included diarrhea or loose stools (7%), nausea (5%), vomiting (2%), and vaginitis (2%) (unpublished data, Pfizer, Inc., 1996).

Some macrolides can inhibit drug metabolism in the liver by complex formation and inactivation of the drug-oxidizing enzyme cytochrome P450.[17] For example, erythromycin has been associated with clinically important interactions with concomitantly administered drugs such as theophylline and carbamazepine. Azithromycin does not form covalent chemical complexes with cytochrome P450 enzymes and does not demonstrate metabolic drug interactions with theophylline, carbamazepine, or terfenadine.[18–20]

### ANTIMICROBIAL SPECTRUM

Azithromycin is highly active against erythromycin-susceptible, gram-positive organisms because of a common mechanism of action.[9] In recent surveys of bacterial pathogens in the United States, the rates of susceptibility to azithromycin and erythromycin were virtually identical for clinical isolates of *Streptococcus pneumoniae*, *S. pyogenes*, *S. agalactiae*, and methicillin-susceptible *Staphylococcus aureus*.[21,22]

Azithromycin is substantially more potent than other macrolides against several clinically important gram-negative pathogens, including *Haemophilus influenzae* and *Moraxella catarrhalis* as well as *Mycoplasma pneumoniae*.[23–25]

In vitro studies have shown that azithromycin and erythromycin have comparable activity against *C. trachomatis*.[13,26,27] The MICs for azithromycin ranged from 0.03 to 0.5 μg/ml in these studies, compared with 0.03 to 1.0 μg/ml for erythromycin.

### CLINICAL APPLICATIONS

The approved indications and dosage schedules for use of azithromycin are summarized in Table 1.

Recent clinical trials[28–30] have shown that azithromycin is effective and well tolerated when administered as a single dose (1 g) in patients with uncomplicated urethritis or cervicitis due to *C. trachomatis*.

In a randomized, comparative study, men attending a sexually transmitted disease (STD) clinic who had positive cultures for *C. trachomatis* received either a single 1-g dose of azithromycin or doxycycline, 100 mg twice daily for 7 days.[28] After 4 weeks, the chlamydial cultures were negative for 43 of 44 patients given azithromycin and 48 of 49 patients given doxycycline. Azithromycin was very well tolerated.

A recent double-blind, multiclinic trial compared single-dose azithromycin with the standard 7-day doxycycline therapy in men with symptomatic nongonococcal urethritis.[29] The cumulative clinical cure rate was 81% among azithromycin recipients and 77% for doxycycline recipients. The cure rates were comparable when patients were stratified by the presence or absence of infection with *C. trachomatis* prior to therapy. Among those with positive baseline cultures, the overall microbiologic cure rates were 83% [95% confidence interval (CI), 65–94%] for azithromycin-treated patients and 90% (95% CI, 68–98%) for doxycycline-treated patients. Gastrointestinal side effects, the most common adverse experiences with both azithromycin and doxycycline, occurred in 19% and 26% of patients, respectively.

In a multicenter trial,[30] 299 women and 158 men with uncomplicated genital infections (urethritis or cervicitis) and antigen tests positive for *C. trachomatis* were randomly assigned to receive oral doses of either azithromycin (1 g once) or doxycycline (100 mg orally twice daily for 7 days). Only the patients subsequently determined to have cultures showing *C. trachomatis* at baseline were evaluated. Bacteriologic eradication was achieved in 96% of the evaluable patients given single-dose azithromycin, compared with 98% of those treated with doxycycline. Among the patients evaluated 21–35 days after treatment began, the eradication rates were 100% with azithromycin and 99% with doxycycline.

Treatment-related side effects were reported in 17% of azithromycin recipients and in 20% of doxycycline recipients. Gastrointestinal symptoms were the most common side effects in each group. Except for 1 azithromycin-treated patient with nausea, the symptoms were mild or moderate in severity.[30]

The efficacy and safety of azithromycin in pregnant women with chlamydial cervicitis have been compared with those of erythromycin.[31] Patients were randomly assigned to receive erythromycin or azithromycin and requested to complete questionnaires identifying adverse events. The cure rates were similar with both treatments. All patients in the erythromycin group, however, reported 2 or more gastrointestinal side effects, compared with none in the azithromycin group. In addition, 5 of 15 erythromycin-treated patients were intolerant of the 500-mg 4-times-daily dosage, requiring a reduction to 250 mg to complete the treatment course. No azithromycin-treated patients failed to tolerate the single 1-g oral dose. These findings suggested that azithromycin is an effective alternative to erythromycin in pregnant patients who are intolerant of erythromycin or noncompliant because of gastrointestinal side effects.

In randomized, multicenter trials in adults with community-acquired respiratory-tract infections,[32–34] azithromycin administered in a 5-day dosage regimen was compared with the standard 10-day regimens of other agents. In all studies, the clinical and bacteriologic efficacy of azithromycin was similar to that of the comparative agents. The infections included acute bacterial exacerbation of chronic obstructive pulmonary disease (chronic bronchitis), mild pneumonia suitable for outpatient oral therapy, and acute pharyngitis or tonsillitis (Table 1).

A multicenter trial in patients with uncomplicated skin and skin-structure infections—predominantly cellulitis or abscesses—showed that azithromycin administered once daily for 5 days was effective clinically and bacteriologically.[35] The strains of *S. aureus* and *S. pyogenes* were the most common bacterial isolates.

### COST

For the treatment of genital chlamydial infections, azithromycin (1 g once) is more cost-effective than the 7-day regimens of doxycycline.[36,37] In the treatment of indicated respiratory-tract and skin and skin-structure infections, 5-day azithromycin therapy has among the lowest cost per treatment of any branded antibiotic.[38] The cost comparisons among antimicrobial agents should consider the expected rates of compliance and expenditures for side effects or drug interactions in addition to drug-acquisition costs.[38,39] A lack of compliance can lead to treatment failure and an increase in the cost of therapy.[40] Further, adverse effects may increase the treatment costs regardless of any effect on compliance.[38]

Compliance is particularly important in the treatment of chlamydial STDs because an asymptomatic infection, which is common, often involves a sexual partner who may be insufficiently motivated to seek medical attention or receive therapy.[5] Among patients treated with the standard 7-day regimens at an STD clinic, compliance has been estimated at 60%.[41] Additional studies are required to determine whether noncompliance with the 7-day doxycycline treatment results in a high rate of therapeutic failure.[42] Single-dose therapy with azithromycin would most likely ensure a 100% rate of compliance.[30]

### CONCLUSIONS

Azithromycin, the first member of the azalide subclass of macrolide antibiotics, is distinguished from other macrolides by its extensive penetration into intracellular and interstitial tissue compartments. Although it shares the gram-positive activity of erythromycin, azithromycin is substantially more potent against gram-negative organisms. The prolonged half-life in serum and tissues allows a 5-day, once-daily dosage schedule in the treatment of indicated respiratory-tract and skin and skin-structure infections (unpublished data, Pfizer, Inc., 1996).

Azithromycin, 1 g orally in a single dose, has been recommended by the CDC for the treatment of chlamydial infections, along with the previously recommended regimen of doxycycline, 100 mg orally twice daily for 7 days.[42] Ofloxacin, 300 mg orally twice daily for 7 days, and the older erythromycin regimens are alternatives. A single dose of azithromycin is less expensive than a 7-day regimen of doxycycline or ofloxacin.

Azithromycin has demonstrated better gastrointestinal tolerability than erythromycin.[16] In addition, azithromycin, a pregnancy category-B drug, may be a valid alternative to erythromycin in pregnant patients (unpublished data, Pfizer, Inc., 1996).[31]

### REFERENCES

1. Sanders LL, Harrison HR, Washington AE: Treatment of sexually transmitted chlamydial infections. JAMA 255:1750–1756, 1986.
2. Pruessner HT, Hansel NK, Griffiths M: Diagnosis and treatment of chlamydial infections. Am Fam Phys 34:81–92, 1986.
3. Toomey KE, Barnes RC: Treatment of *Chlamydia trachomatis* genital infection. Rev Infect Dis 12(Suppl 6):S645–S655, 1990.
4. Washington AE, Katz P: Cost of and payment source for pelvic inflammatory disease: Trends and projections, 1983 through 2000. JAMA 266:2565–2569, 1991.
5. Stamm WE: Azithromycin in the treatment of uncomplicated genital chlamydial infections. Am J Med 91(Suppl 3A):19S–22S, 1991.
6. Majeroni BA: Chlamydial cervicitis: Complications and new treatment options. Am Fam Phys 49:1825–1829, 1994.
7. Schentag JJ, Ballow CH: Tissue-directed pharmacokinetics. Am J Med 91(Suppl 3A):5S–11S, 1991.
8. Bahal N, Nahata MC: The new macrolide antibiotics: Azithromycin, clarithromycin, dirithromycin, and roxithromycin. Ann Pharmacother 26:46–55, 1992.
9. Retsema J, Girard A, Schelkly W, et al.: Spectrum and mode of action of azithromycin (CP-62,993), a new 15-membered-ring macrolide with improved potency against gram-negative organisms. Antimicrob Agents Chemother 31:1939–1947, 1987.
10. Piscitelli SC, Danziger LH, Rodvold KA: Clarithromycin and azithromycin: New macrolide antibiotics. Clin Pharm 11:137–152, 1992.
11. Neu HC: New macrolide antibiotics: Azithromycin and clarithromycin. Ann Intern Med 116:517–519, 1992.
12. Foulds G, Shepard RM, Johnson RB: The pharmacokinetics of azithromycin in human serum and tissues. J Antimicrob Chemother 25(Suppl A):73–82, 1990.
13. Scieux C, Bianchi A, Chappey B, Vassias I, Pérol Y: In-vitro activity of azithromycin against *Chlamydia trachomatis*. J Antimicrob Chemother 25(Suppl A):7–10, 1990.

14. Krohn K: Gynaecological tissue levels of azithromycin. Eur J Clin Microbiol Infect Dis 10:864–868, 1991.
15. Gladue RP, Bright GM, Isaacson RE, Newborg MF: In vitro and in vivo uptake of azithromycin (CP-62,993) by phagocytic cells: Possible mechanism of delivery and release at sites of infection. Antimicrob Agents Chemother 33:277–282, 1989.
16. Hopkins SJ: Clinical toleration and safety of azithromycin in adults and children. Rev Contemp Pharmacother 5:383–389, 1994.
17. Periti P, Mazzei T, Mini E, Novelli A: Pharmacokinetic drug interactions of macrolides. Clin Pharmacokinet 23: 106–131, 1992.
18. Honig PK, Wortham DC, Zamani K, Cantilena LR: Comparison of the effect of the macrolide antibiotics erythromycin, clarithromycin and azithromycin on terfenadine steady-state pharmacokinetics and electrocardiographic parameters. Drug Invest 7:148–156, 1994.
19. Gardner MJ, Coates PE, Hilligoss DM, Henry EB: Lack of effect of azithromycin (AZ) on the pharmacokinetics (PK) of theophylline (T) in man. Abstract 410. In: Program and Abstracts of the Mediterranean Congress of Chemotherapy, Athens, 1992.
20. Rapeport WG, Dewland PM, Muirhead DC, Forster PL: Lack of an interaction between azithromycin and carbamazepine. Br J Clin Pharmacol 33:551P, 1992.
21. Fass RJ: Erythromycin, clarithromycin, and azithromycin: Use of frequency distribution curves, scattergrams, and regression analyses to compare in vitro activities and describe cross-resistance. Antimicrob Agents Chemother 37:2080–2086, 1993.
22. Barry AL, Fuchs PC, Brown SD: Relative potencies of azithromycin, clarithromycin and five other orally administered antibiotics. J Antimicrob Chemother 35:552–555, 1995.
23. Hardy DJ, Hensey DM, Beyer JM, Vojtko C, McDonald EJ, Fernandes PB: Comparative in vitro activities of new 14-, 15-, and 16-membered macrolides. Antimicrob Agents Chemother 32:1710–1719, 1988.
24. Brown SD, Burton P, Rich T, Bouchillon SK, Yee YC: Susceptibility surveillance of U.S. respiratory pathogen isolates to newer macrolide and azalide antibiotics. Abstract A32. Presented at the 94th General Meeting of the American Society for Microbiology, Las Vegas, 1994.
25. Felmingham D, Robbins MJ, Sanghrajka M, Leakey A, Ridgway GL: The in vitro activity of 14-, 15-, and 16-membered macrolides against *Staphylococcus* spp., *Legionella* spp., *Mycoplasma* spp. and *Ureaplasma urealyticum.* Drugs Exp Clin Res 17:91–99, 1991.
26. Slaney L, Chubb H, Ronald A, Brunham R: In-vitro activity of azithromycin, erythromycin, ciprofloxacin and norfloxacin against *Neisseria gonorrhoeae, Haemophilus ducreyi*, and *Chlamydia trachomatis.* J Antimicrob Chemother 25(Suppl A):1–5, 1990.
27. Welsh LE, Gaydos CA, Quinn TC: In vitro evaluation of activities of azithromycin, erythromycin, and tetracycline against *Chlamydia trachomatis* and *Chlamydia pneumoniae.* Antimicrob Agents Chemother 36:291–294, 1992.
28. Steingrimsson O, Olafsson JH, Thorarinsson H, Ryan RW, Johnson RB, Tilton RC: Azithromycin in the treatment of sexually transmitted disease. J Antimicrob Chemother 25(Suppl A):109–114, 1990.
29. Stamm WE, Hicks CB, Martin DH, et al.: Azithromycin for empirical treatment of the nongonococcal urethritis syndrome in men. JAMA 274:545–549, 1995.
30. Martin DH, Mroczkowski TF, Dalu ZA, et al.: A controlled trial of a single dose of azithromycin for the treatment of chlamydial urethritis and cervicitis. N Engl J Med 327:921–925, 1992.
31. Bush MR, Rosa C: Azithromycin and erythromycin in the treatment of cervical chlamydial infection during pregnancy. Obstet Gynecol 84:61–63, 1994.
32. Dark D: Azithromycin versus cefaclor in the treatment of acute exacerbations of chronic obstructive pulmonary disease. Curr Ther Res 53:203–211, 1993.
33. Kinasewitz G, Wood RG: Azithromycin versus cefaclor in the treatment of acute bacterial pneumonia. Eur J Clin Microbiol Infect Dis 10:872–877, 1991.
34. Hooton TM: A comparison of azithromycin and penicillin V for the treatment of streptococcal pharyngitis. Am J Med 91(Suppl 3A):23S–26S, 1991.
35. Mallory SB: Azithromycin compared with cephalexin in the treatment of skin and skin structure infections. Am J Med 91(Suppl 3A):36S–39S, 1991.
36. Haddix AC, Hillis SD, Kassler WJ: The cost effectiveness of azithromycin for *Chlamydia trachomatis* infections in women. Sex Transm Dis 22:274–280, 1995.
37. Magid D, Douglas JM, Jr, Schwartz JS: Doxycycline compared with azithromycin for treating women with genital *Chlamydia trachomatis* infections: An incremental cost-effectiveness analysis. Ann Intern Med 124:389–399, 1996.
38. Nightingale CH, Belliveau PP, Quintiliani R: Cost issues and considerations when choosing antimicrobial agents. Infect Dis Clin Pract 3:8–11, 1994.
39. Hamilton RA, Gordon T: Incidence and cost of hospital admissions secondary to drug interactions involving theophylline. Ann Pharmacother 26:1507–1511, 1992.
40. Davey P, Parker S: Cost effectiveness of once-daily oral antimicrobial therapy. J Clin Pharmacol 32:706–710, 1992.
41. Katz BP, Zwickl BW, Caine VA, Jones RB: Compliance with antibiotic therapy for *Chlamydia trachomatis* and *Neisseria gonorrhoeae.* Sex Transm Dis 19:351–354, 1992.
42. Levine WC, Berg AO, Johnson RE, et al.: Development of sexually transmitted diseases treatment guidelines, 1993: New methods, recommendations, and research priorities. Sex Transm Dis 21(Suppl):S96–S101, 1994.
